# Correlation of Homocysteine Level and Age in Patients with Ischemic Stroke

**DOI:** 10.7759/cureus.7785

**Published:** 2020-04-22

**Authors:** Muhammad Omer Sultan, Umar Farooque, Rafay Javed, Muhammad I Khan, Sundas Karimi, Rukhsana Abdul Sattar, Omer Cheema

**Affiliations:** 1 Internal Medicine, Jinnah Postgraduate Medical Center, Karachi, PAK; 2 Neurology, Dow Medical College, Karachi, PAK; 3 Internal Medicine, Jinnah Hospital, Allama Iqbal Medical College, Lahore, PAK; 4 General Surgery, Combined Military Hospital, Karachi, PAK; 5 Internal Medicine, Jinnah Postgraduate Medical Centre, Karachi, PAK; 6 Internal Medicine, Dow University of Health Sciences, Karachi, PAK

**Keywords:** homocysteine level, correlation, ischemic stroke, age, humans, diabetes mellitus, hypertension

## Abstract

Introduction

Hyperhomocysteinemia is associated with atherosclerosis, as it can be seen in inborn errors of methionine metabolism. Likewise, many studies have also reported more modest increases in serum homocysteine levels in other atherosclerotic disorders like cardiovascular disease and all types of stroke with a positive correlation with age. But overall literature is controversial. Therefore, this study is being conducted to further investigate the relationship between homocysteine ​​levels and age in patients, especially those with ischemic stroke.

Material and methods

This cross-sectional study is conducted at a major hospital in Karachi in which all patients with ischemic stroke, diagnosed within 24 hours on CT, and age 40-75 years of both genders were enrolled for six months. Other demographics were also noted like gender, smoking status, and comorbidities (diabetes mellitus [DM], hypertension [HTN]). The homocysteine level was also checked by collecting non-fasting blood. Vitamin B12 level was not checked. The age, weight, height, body mass index (BMI), and homocysteine level’s means and standard deviations and the gender, DM, hypertension, and smoking status frequencies and percentages were calculated. The correlation coefficient of homocysteine level and age was also calculated. Stratification was done to see the effects of gender, BMI, DM, and HTN on homocysteine levels by applying the chi-square test.

Results

The mean age of the patients was 55.60 ± 11.45 years. Gender distribution showed that 111 (62.40%) patients were male, and 67 (37.60%) patients were female. Diabetic, hypertensive, and smoking status of the patients was 58 (32.60%), 96 (53.90%), and 53 (29.80%), respectively. The mean homocysteine level was 14.61, with a standard deviation of 1.47. Pearson’s correlation test showed that there is no statistically significant correlation between homocysteine levels and age. But a significant linear relationship was found of homocysteine levels with DM and HTN.

Conclusion

Further investigation of the relationship of homocysteine ​​levels with age, diabetes mellitus, and hypertension, and the role of homocysteine as a risk factor for ischemic stroke should be carried out on a larger scale to prove its accuracy. The benefits of screening for homocysteine ​​levels also need to be studied in the elderly, especially those with diabetes mellitus and hypertension, which can lead to timely prevention of strokes and ischemic heart disease with vitamin B supplements, and other appropriate interventions.

## Introduction

Homocysteine is a prothrombotic factor that affects coagulation and fibrinolytic cascades [1]. It also causes endothelial injury through direct toxicity and apoptosis which results in endothelial dysfunction causing impaired endothelium-dependent dilatation of blood vessels combined with a proinflammatory and proatherosclerotic endothelial phenotype [2, 3].

That’s why inborn errors of methionine metabolism, associated with very high levels of homocysteine, cause atherosclerosis [4]. But atherosclerotic diseases like coronary artery disease and stroke, without underlying hereditary disorders, can also have slightly increased serum homocysteine levels. In fact, some meta-analyses have shown independent stratified associations of homocysteine levels with stroke and ischemic heart disease [5, 6].

Stroke is a heterogeneous disease and different stroke subtypes have different pathophysiological mechanisms. Homocysteine ​​has been studied in stroke subtypes in various Asian and European populations, and most studies show that all stroke subtypes have high homocysteine ​​levels compared to controls [7-13]. Homocysteine levels were elevated in stroke patients (14.3±8.8 µmol/L) compared to controls (11.8±5.7 µmol/L). Homocysteine concentrations were separately elevated in both ischemic (14.3±8.9 µmol/ L) and hemorrhagic (14.5±8.7 µmol/L) strokes compared to controls. Furthermore, homocysteine was positively correlated with age in both ischemic and hemorrhagic strokes (r=0.211) and it was also associated with premature ischemic stroke in young patients [14, 15].

On robust search, we found that no local data on the correlation of homocysteine level and age in patients with ischemic stroke is available and there is scarce literature available internationally. Also, the data review showed that there is a lot of controversy about the importance of homocysteine ​​as a risk factor for CVD and stroke [16]. Therefore, the present study is designed with the view to generate local data and to estimate the strength of association of homocysteine level and age in patients with ischemic stroke.

## Materials and methods

Study design and sampling

The cross-sectional study took place at Jinnah Postgraduate Medical Center, Karachi, from 6/7/2013 to 12/7/2013 (during six months). Non-probability consecutive sampling technique was applied. The sample size was calculated, taking into account that the null hypothesis with 5% type I and 10% type II errors, homocysteine level, and age correlation (r)^15^ =0.211, the sample size came out to be 178 patients with ischemic stroke. Inclusion criteria are age 40-75 years, either gender, patients with ischemic stroke diagnosed within 24 hours on CT. Exclusion criteria included patients with hemorrhagic stroke, recurrent ischemic strokes, subcortical infarction >15 mm diameter, cortical infarction, carotid, vertebral, or intracranial artery stenosis >50% on CT, and potential cardiac source of embolism.

Data collection

Ischemic stroke patients, diagnosed as specified in the operational definition meeting the inclusion criteria, admitted through the emergency department, were enrolled in the study. Prior to enrolment, the pros and cons of the study were explained, and informed consent was obtained. Non-fasting blood was collected and sent to the institutional laboratory for the homocysteine level analysis. Patients demographics like age, gender, smoking status, and comorbidities (diabetes mellitus, hypertension) were asked by the researcher and entered in the questionnaire. Vitamin B12 level was not checked.

Data analysis

Data was entered and analyzed on SPSS version 17 (SPSS Statistics, Chicago, US). Mean and the standard deviation were calculated for age, weight, height, body mass index (BMI), and homocysteine level. Frequency and percentage for gender, diabetes mellitus (DM), hypertension (HTN) and smoking status, and correlation coefficient of homocysteine level and age were also calculated. Effect modifiers like gender, BMI, DM, and HTN were controlled through stratification, the Chi-square test was applied, and p-value ≤0.05 was considered as significant.

## Results

**Figure 1:**
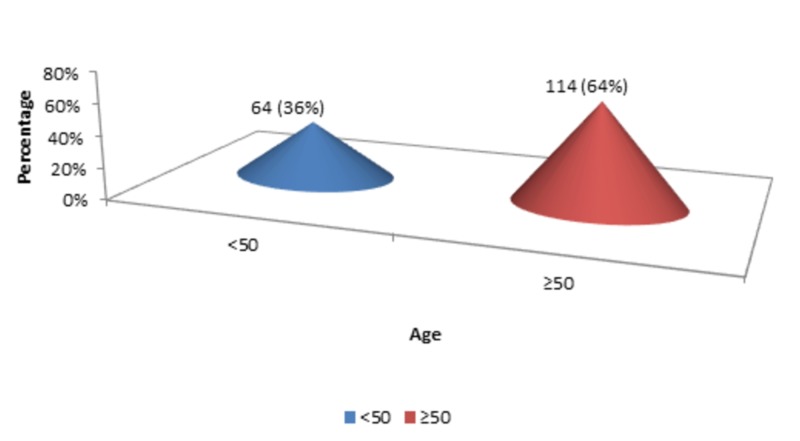
Age groups

The mean age of the patients was 55.60 years, with a standard deviation of 11.45 years (n=178). The minimum age of the patients was 40 years, while the maximum age of the patients was 75 years. There were 64 (36%) patients with <50 years of age, while 114 (64%) patients with ≥50 years of age, as shown in Figure 1.

**Figure 2:**
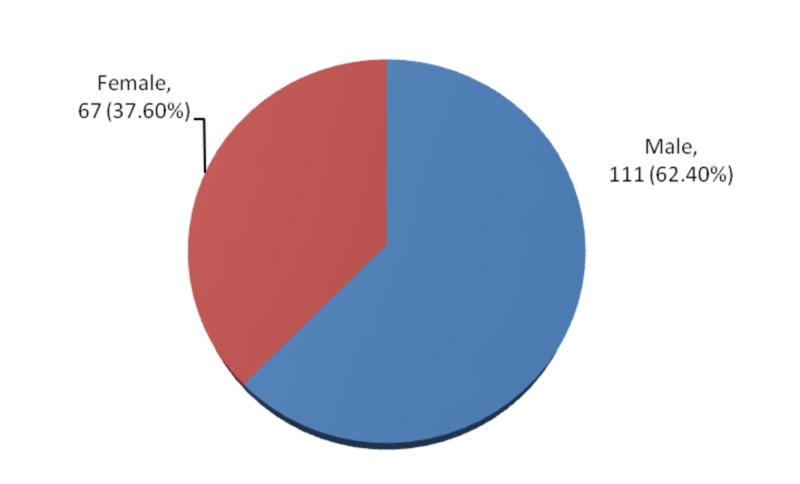
Gender distribution

The gender distribution showed 111 (62.40%) male and 67 (37.60%) female, as shown in Figure 2.

The mean weight was 66.92 kg, with a standard deviation of 5.71 kg (n=178). The minimum weight of the patients was 56 kg, while the maximum weight of the patients was 83 kg. The mean height was 1.58 meters, with a standard deviation of 0.04 meters (n=178). The minimum height of the patients was 1.50 meters, while the maximum height of the patients was 1.74 meters. 

**Figure 3:**
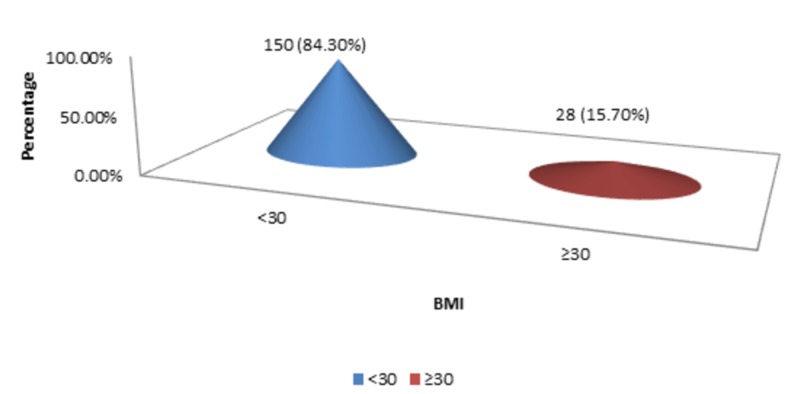
BMI BMI - body mass index

The mean BMI was 25.01 kg/m^2, ^with a standard deviation of 5.05 kg/m^2^ (n=178). The minimum BMI of the patients was 24 kg/m^2,^ while the maximum BMI of the patients was 33 kg/m^2^. There were 150 (84.30%) patients with <30 kg/m^2^ BMI, and 28 (15.70%) patients were present with ≥30 kg/m^2^ BMI as shown in Figure 3.

**Figure 4:**
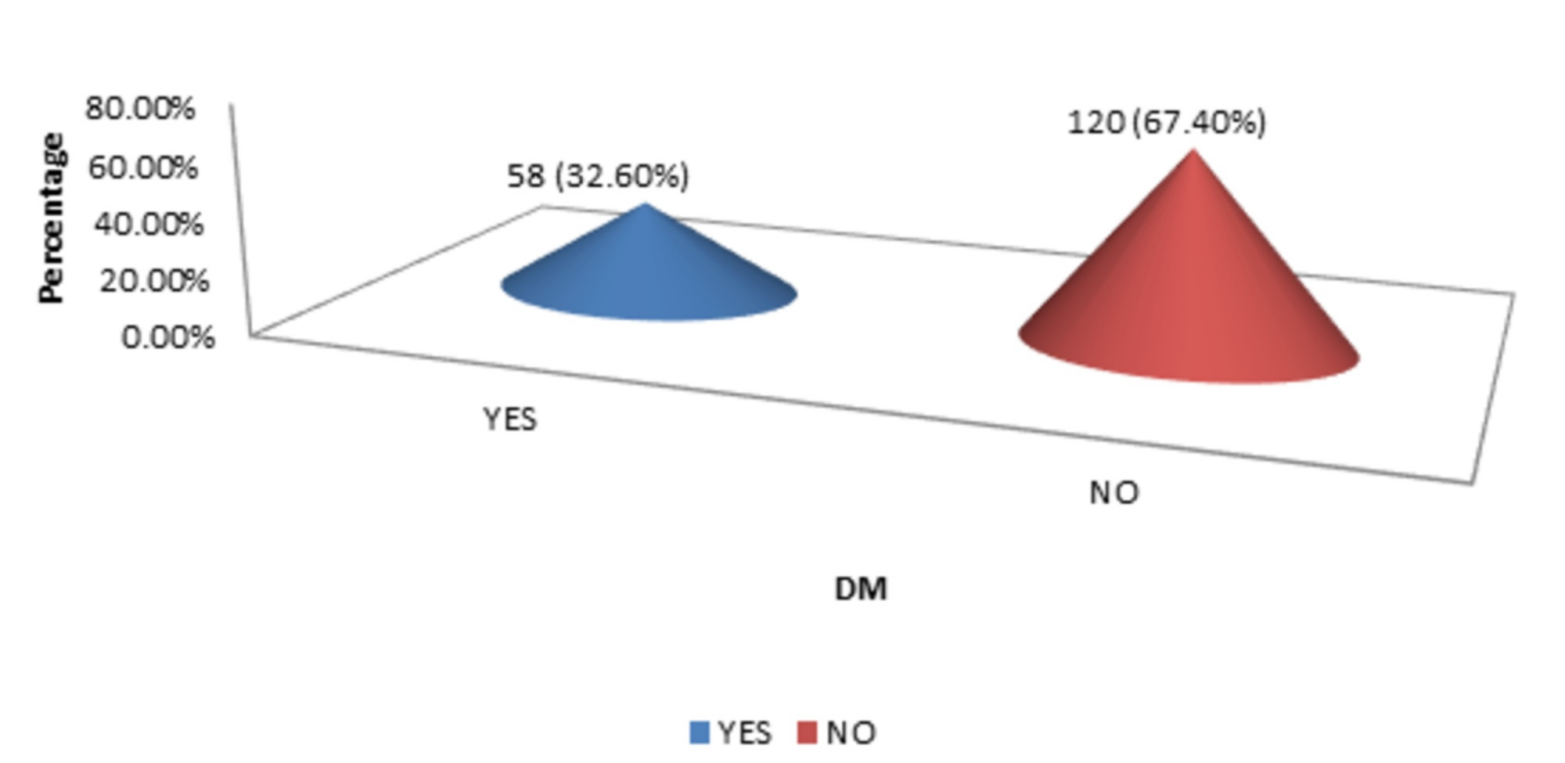
DM DM - diabetes mellitus

**Figure 5:**
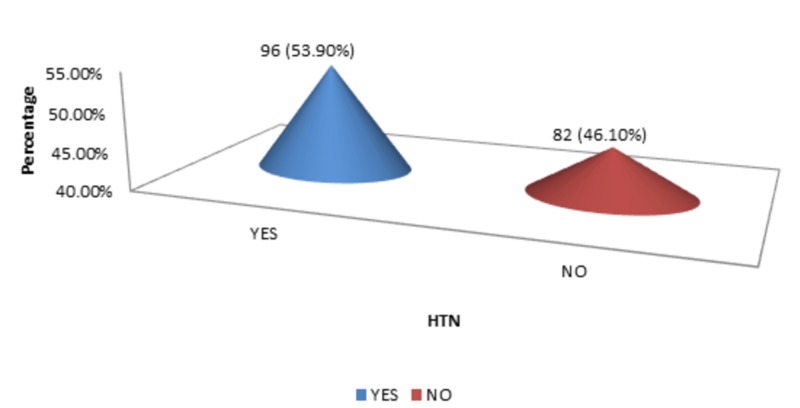
HTN HTN - hypertension

**Figure 6:**
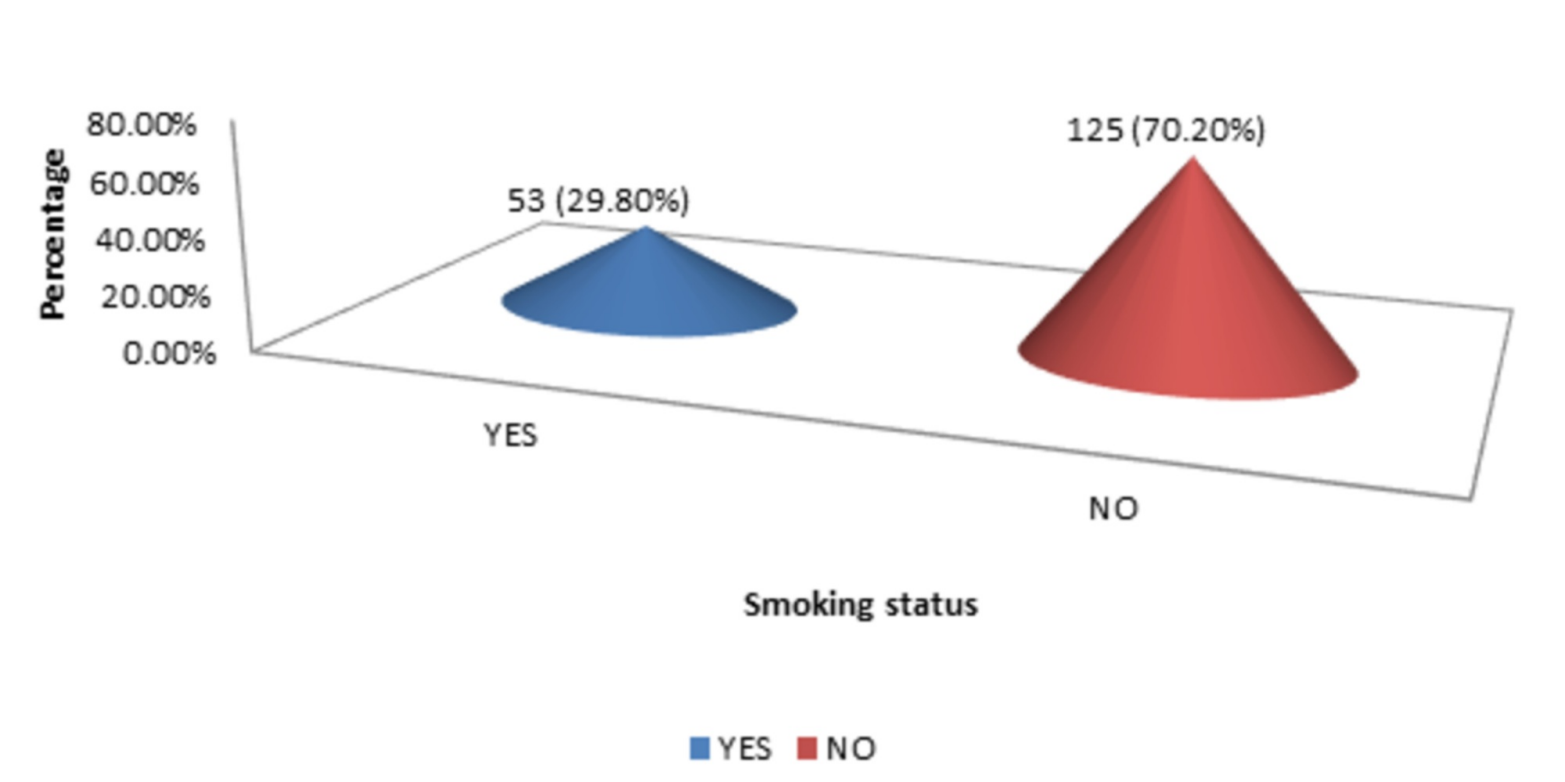
Smoking status

The DM, HTN, and smoking status were 58 (32.60%), 96 (53.90%), and 53 (29.80%), respectively, as shown in Figure 4-6.

**Figure 7:**
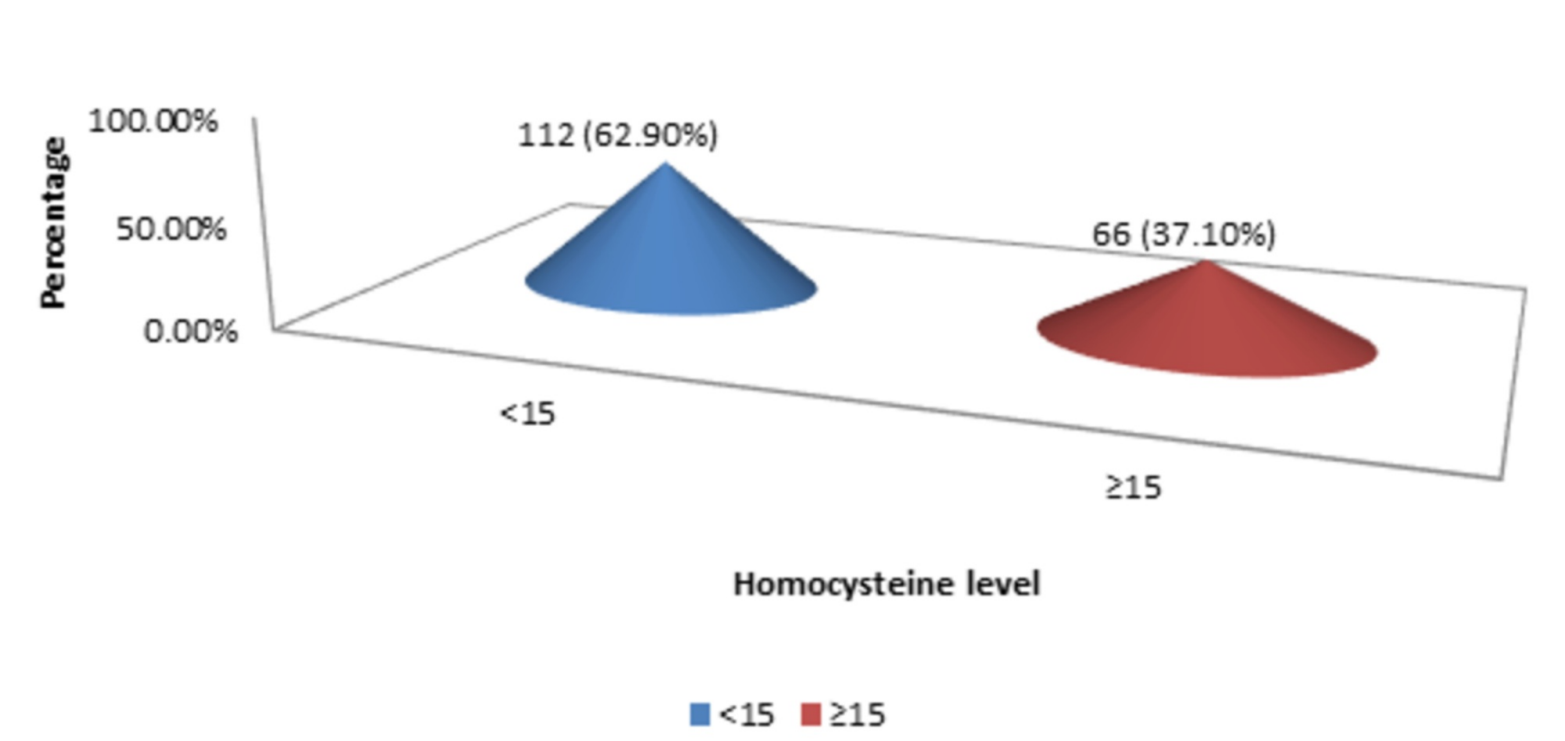
Homocysteine level

The mean homocysteine level was 14.61 micromol/l with a standard deviation of 1.47 (n=178; min 12, max 17). Most of the patients were presented with <15 homocysteine level, i.e., 112 (62.90%), as shown in Figure 7.

A statistically insignificant correlation among homocysteine level and age was calculated by applying Pearson’s correlation test, as shown in Table 1.

**Table 1:** Correlation of homocysteine level with age (n=178)

		Age	Homocysteine level
Age	Pearson’s correlation	1	0.21
	p-value		0.778
Homocysteine level	Pearson’s correlation	0.21	1
	p-value	0.778	

Stratification was done to see the effects of gender, BMI, DM, and HTN on homocysteine levels. Chi-square test was applied, and statistically sufficient evidence of significant relationship was observed in DM (p=0.002) and HTN (p=0.008), as shown in Tables 2-5.

**Table 2:** Comparison of homocysteine level with gender (n=178)

Gender	Homocysteine level		Total	p-value
	<15	≥15		
Male	66 (59.5)	45 (40.5)	111 (100)	0.263
Female	46 (68.7)	21 (31.3)	67 (100)	
Total	112 (62.9)	66 (37.1)	178 (100)	

**Table 3:** Comparison of homocysteine level with BMI (n=178) BMI - body mass index

BMI	Homocysteine level		Total	p-value
	<15	≥15		
<30	97 (64.7)	53 (35.3)	150 (100)	0.291
≥30	15 (53.6)	13 (46.4)	28 (100)	
Total	112 (62.9)	66 (37.1)	178 (100)	

**Table 4:** Comparison of homocysteine level with DM (n=178) DM - diabetes mellitus

DM	Homocysteine level		Total	p-value
	<15	≥15		
Yes	46 (79.3)	12 (20.7)	58 (100)	0.002
No	66 (55.)	54 (45)	120 (100)	
Total	112 (62.9)	66 (37.1)	178 (100)	

**Table 5:** Comparison of homocysteine level with HTN (n=178) HTN - hypertension

HTN	Homocysteine level		Total	p-value
	<15	≥15		
Yes	69 (71.9)	27 (28.1)	96(100)	0.008
No	43 (52.4)	39 (47.6)	82 (100)	
Total	112 (62.9)	66 (37.1)	178 (100)	

## Discussion

This study showed that there is no significant correlation between serum homocysteine levels and the age of patients with ischemic stroke as per Pearson's correlation test, with a mean homocysteine level of 14.61 and a standard deviation of 1.47 but diabetes mellitus and hypertension are significantly associated with increased homocysteine levels. 

Dutch researchers have found that high homocysteine ​​levels are associated with significantly higher risks of stroke and heart attack. Their study included 7,983 elderly patients whose homocysteine ​​levels were measured in July 1993. In December 1994, 120 had a stroke, and 104 had a heart attack. Researchers have found that the risk of stroke and heart attack increases linearly with increasing homocysteine ​​levels, so an increase of 1 mmol/l at homocysteine ​​levels is associated with a 6-7% increase in the risk of stroke or heart attack. Participants in the study with the highest homocysteine ​​levels had twice the risk of stroke compared with participants with the lowest homocysteine ​​levels after adjusting for age, sex, smoking, high blood pressure, cholesterol, and diabetes. The increased risk of stroke is seen in both ischemic and hemorrhagic strokes and is increased in the presence of high blood pressure. It is interesting to note that the annual stroke rate in the group was 1.0% [17].

Previous research also showed that patients with homocysteine ​​levels greater than 14.2 mmol/l had a stroke rate of 82% higher than participants with a level of 9.25 mmol/l or less after ten years of study. Interestingly, the mean annual stroke rate among study participants (mean age of 70 years) was 0.8% [18].

Swiss researchers have observed a strong relationship between coronary artery disease and high homocysteine ​​levels. They found that an increase of only 5 mmol/l was in accordance with an increased risk of coronary artery disease by 60% in men and 80% in women. They also found that homocysteine ​​levels in 631 patients undergoing angiography increased linearly from 9.2 mmol/l in patients without coronary artery disease to 12.4 mmol/l in patients with the three-vessel disease [19].

Taiwanese and Italian researchers have found a strong relationship between high homocysteine ​​levels and the presence of endothelial dysfunction and atherosclerosis. Not surprisingly, they also reported that high protein foods would significantly increase homocysteine ​​levels. Fortunately, they also found that supplementing the five-week vitamin B cocktail significantly reduced the increase in protein-induced hyperhomocysteinemia [20, 21].

Researchers at the Boston University School of Medicine report that people with homocysteine ​​levels above 14 mmol/l are almost twice as likely to develop Alzheimer's disease as people with lower levels. They also found that an increase in homocysteine ​​levels of 5 mmol/l was associated with a 40% increase in Alzheimer's disease risk [22]. This neurodegenerative damage of homocysteine can be due to its direct neurotoxic effect and its role in endothelial injury [23].

## Conclusions

The increase of homocysteine ​​level with age, its role ​​as a risk factor for stroke, coronary artery disease, and neurodegenerative diseases such as Alzheimer's, and its association with diabetes mellitus and hypertension still needs more comprehensive analysis. Studies are also needed to determine the benefits of using homocysteine ​​levels as a screening tool in the elderly for strokes, ischemic heart disease, and neurodegenerative diseases, especially in diabetics and hypertensives, so that strategies can be devised to prevent them earlier with food supplements such as folic acid, vitamin B6 and vitamin B12, and other timely interventions.
